# Human and Animal Motion Tracking Using Inertial Sensors

**DOI:** 10.3390/s20216074

**Published:** 2020-10-26

**Authors:** Frédéric Marin

**Affiliations:** Centre of Excellence for Human and Animal Movement Biomechanics (CoEMoB), Laboratoire de BioMécanique et BioIngénierie (UMR CNRS 7338), Université de Technologie de Compiègne (UTC), Alliance Sorbonne Université, 60200 Compiègne, France; frederic.marin@utc.fr; Tel.: +33-3-4423-4423

**Keywords:** inertial sensors, Inertial Mouvement Unit (IMU), motion capture, motion analysis, biomechanics

## Abstract

Motion is key to health and wellbeing, something we are particularly aware of in times of lockdowns and restrictions on movement. Considering the motion of humans and animals as a biomarker of the performance of the neuro-musculoskeletal system, its analysis covers a large array of research fields, such as sports, equine science and clinical applications, but also innovative methods and workplace analysis. In this Special Issue of Sensors, we focused on human and animal motion-tracking using inertial sensors. Ten research and two review papers, mainly on human movement, but also on the locomotion of the horse, were selected. The selection of articles in this Special Issue aims to display current innovative approaches exploring hardware and software solutions deriving from inertial sensors related to motion capture and analysis. The selected sample shows that the versatility and pervasiveness of inertial sensors has great potential for the years to come, as, for now, limitations and room for improvement still remain.

## 1. Introduction

The motion of humans or animals is a biomarker of the performance of the neuro-musculoskeletal system. Consequently, if the neuro-musculoskeletal system works “well”, the movement performed by the subject is smooth and efficient too. This being said, the real question is how to quantify the right amount of “well”. For almost 200 years now, researchers have searched for technologies and methods to “measure” this motion and to determine relevant metrics for it [1]. Human and animal motion capture became a vibrant field for technological innovations. Over time, motion capture systems came to be more accurate and reliable, and after the mid-20th-century, motion analysis grew to be a relevant method for clinical diagnosis, follow-up, sports and ergonomics applications.

In the early 21st century, improvements in the technology of inertial sensors combining accelerometers and gyrometers, completed by magnetometers, pressure sensors, etc., allowed for new perspectives as far as motion capture and analysis of humans and animals are concerned. Due to the versatility of inertial sensors, measurement sessions can easily be conducted outside the laboratory, i.e., at the workplace or in field studies. These “ecologic” sessions offer a new perspective to investigate human movement in relation with the environment. They allow both for sessions of either a very short duration, such as shock and crash situations, and for sessions lasting several days, i.e., monitoring physical activity. Inertial sensors are used either as single sensors or inertial sensor networks to record kinematics or dynamics of single anatomical segments, the upper and lower limbs, or even the full body. However, as in every other emerging technology, once the excitement related to the novelty has passed, limitations of use and best practices appear, calling for rational and efficient applications. This is where we are now, and this Special Issue would like to display innovative work exploring hardware and software solutions deriving from inertial sensors related to human or animal motion capture and analysis. 

## 2. Contributions

First, there are two review papers concerning sensor-to-segment calibration [2] and the use of inertial sensors for 6-min walk test quantification [3]. 

The first systematic review [2] looks into one of the redundant issues in the use of inertial sensors for motion capture. It is about sensor-to-segment calibration for the lower limb. Actually, the classic method of human and animal motion analysis investigates the kinematics of anatomical segment. Thanks to the hardware architecture and specific orientation calculation, inertial sensors can provide the kinematics of their own reference frame fully decoupled from the anatomical segment reference frame where the sensor is fixed onto. A calibration is required to define the relationship between the reference frame of the sensor and the one of the anatomical segment. This systematic review gives us an overview of the main approaches used to perform this sensor-to-segment calibration. 

The second systematic review [3] focuses on the use of inertial sensors to monitor the 6-min walk test (6MWT). This exercise is one of the clinical tests to quantify the general health status of a subject. The use of inertial sensors for 6MWT is adapted due to the versatility of the inertial sensors. The counterpart of this flexible use is not only the heterogeneity in types of sensors, methods, sensors localization and parameters, but also in the pathologies targeted. This systematic review helps us to obtain a global vision of the current practice and possible ways to improve the best use of inertial sensors to analyze the 6-min walk test. 

The second set of papers are research papers focusing on sports applications: soccer [4], ski [5], canoe [6] and racket playing [7]. 

To begin with, the first sport-related research paper is dedicated to the hip joint motion analysis of adolescent soccer players using inertial sensors [4]. In addition to performance quantification, such an investigation is also motivated to prevent injuries. This is critical for their later career when playing at a professional elite-level. The authors describe a method to determine the hip joint angles and the external training loads by a metric deduced from accelerometer data. The article compares results of elite-level and recreational soccer players. Beyond the fact, and as anticipated, that the range of motion of the hip and the hip accelerations of the elite-level player are higher than those of recreational players, the authors openly share their difficulties and the limitation for the use of inertial sensors, such as sensor calibration, magnetic field disturbances and sensor biases. Such papers encourage us to use inertial sensors with caution, but also suggest ways for improvement. 

The second sport-related research paper is focused on the use of inertial sensors to classify skiing practice and techniques [5]. In their paper, the authors present four skiing turn styles: snowplow, snowplowstreering, drifting and carving. Data were captured by inertial sensors located at the skiing boots. Using data from 2063 segmented individual turns, 75% were used as a training set for the classifier and 25% as test data to quantify the performance of the classifier. The authors tested three classifier methods: decision tree, random forest and the gradient-boosted decision tree. They demonstrated that the last method gives a slightly better prediction. The authors also detailed the 10 most important variables, which help to differentiate between parallel and non-parallel turns. 

The third sport-related research paper proposed a full method ranging from hardware choices to the processing and capture of full-body motion of athletes during canoeing practice [6]. Therefore the authors detailed the inertial sensor architecture, the fusion algorithm and body segment orientation calculation to capture a 17-rigid-segment model of the rower. For the upper limbs, they compared results with an optical motion tracking. Furthermore, considering that the performance and efficiency of the canoe propulsion is related to the regularity of the stroke cycle, the authors suggested comparing the duration of the stroke cycle between expert and novice rowers. Segmentation of the stroke appears to be an important issue. A machine learning approach was used to classify body kinematics variables according to rowing skill. In their conclusion, the authors insisted on the wearability of their approach. 

The fourth research paper dedicated to sports is about the use of single inertial sensors located at the wrist to count and classify the type of strokes for racket sports, such as badminton and table tennis [7]. The authors worked out a complete solution, presenting a holistic approach including hardware, post-processing of the accelerometer and gyrometer data, classifier methods and an app interface. Their discussion focused on the comparison of the performance of various classifiers. To conclude, the authors even detailed the feasibility of the proposed solution as a potential product for commercial application.

The second set of two research papers is dedicated to equine science, analyzing the horse locomotion phase [8] and estimating the speed per strike [9].

The first concerns the detection of the stance of the horse locomotion and the stride duration of the foot on and off [8]. In their article, the authors presented an inertial sensor solution located at the distal part of the lower limb. They compared four methods based on accelerometer and gyrometer data using a threshold approach and wavelet signal analysis. To compare the performance of these methods, the authors compared their findings with reference data obtained using optical motion capture. In fact, horse locomotion identification by inertial sensors requires certain specifications and methods. It appears that, for this application, inertial sensor motion tracking presents advantages when compared to optical methods. 

The second research paper in relation to equine science concerns the estimation of the speed per strike of the horse [9]. Therefore, the inertial sensor is located in the saddle and a machine learning method is applied to relate the six signals of the accelerometers and the gyrometer to the speed per strike. The data obtained were compared with results of an optical motion capture system, which were used as reference data. The authors presented an original method, confirming that inertial sensors can offer various approaches to extract kinematic parameters.

The third set of two more research papers proposed innovative methods to explore kinematics by inertial sensors. The first one deals with the estimation of the axis of rotation [10] and the other one with the use of inertial sensors in smartphones to identify gait. 

The first research paper with innovative methods proposes determining the orientation of the axis of rotation between two rigid bodies using inertial sensors fixed on these bodies [10]. The authors detail their mathematical formalism. They proposed using raw data of both accelerometers and gyrometers. A cost function is used to minimize and document the kinematic properties of the axis of rotation. In a second step, the authors share their experimental setup of the two rigid segments connected by a hinge joint. The method presented could also be applicable to the functional calibration of human joints. 

The second research paper with innovative methods explores the feasibility of use of inertial sensors of smartphones for walking recognition [11]. In their article, the authors suggest analyzing several metrics of the motion deduced from the inertial sensors of the smartphone using various machine learning techniques to identify the gait of the subject. Therefore, the whole methodology of the postprocessing of smartphone data was detailed. The authors also presented an experimental validation. As additional material, the authors proposed a full dataset of 77 subjects to the scientific community, in order to compare the performance of their method to others. 

Finally, the last set of two research papers concerns industry applications [12,13]. 

The first one is focused on the benefit of the use of a virtual reality system to practice and improve the postural stability of workers working at height [12]. The authors mainly detail the consequence of the practice of specific exercises through virtual reality on the displacement of the center of pressure. In the context of this Special Issue dedicated to inertial sensors, this research paper is interesting as it presents how the orientation of a balance platform is tracked by inertial sensors. In this case, the balance platform has the same function as a joystick. 

The second research paper proposed investigating the estimation of the wrist stiffness during tooling [13]. The goal of the paper is to present a model of a human manipulator performing a polishing task. It aims to track the technique and then to transfer this knowledge to a robot. The present paper tracked motion with an optical motion capture system, which limits the complexity of the task investigated. This very complete paper offers a perspective on the potential use of inertial sensors, which could simplify the motion capture protocol and movement analysis. 
